# A new genus and species of Rhizoecidae (Hemiptera, Sternorryncha, Coccomorpha) associated with *Acropyga yaeyamensis* (Hymenoptera, Formicidae, Formicinae)

**DOI:** 10.3897/zookeys.616.9442

**Published:** 2016-09-12

**Authors:** Hirotaka Tanaka

**Affiliations:** 1 Faculty of Agriculture, Ehime University, Tarumi 3-5-7, Matsuyama, Ehime 790-8566, Japan; 2 Present address: Faculty of Agriculture, Kyushu University, 744 Motooka Nishi-ku, Fukuoka 819-0395, Japan

**Keywords:** Ant trophobiont, ground mealybug, new genus, root mealybug, taxonomy

## Abstract

*Ishigakicoccus* **gen. n.** *shimadai* **sp. n.** is described based on the adult female morphology. This new species was found in the nest of a rare Japanese ant, *Acropyga yaeyamensis* Terayama & Hashimoto, 1996, in Ishigaki Is., Japan. It resembles *Capitisetella migrans* (Green, 1933) and *Pseudorhizoecus proximus* Green, 1933. However, the new species differs from those two species in having small multilocular pores, large 3-5 locular pores on the medial area of ventral abdomen, and two different-sized body setae. This new species is also the first record of a potential trophobiont of *Acropyga yaeyamensis*. A key to Japanese species of Rhizoecidae is also provided.

## Introduction

Rhizoecidae is a family of Coccomorpha (Hemiptera: Sternorryncha) which members mostly live underground, feeding on plant rootlets (Williams 1998), and are known as “root mealybugs” or “ground mealybugs”. The family is composed of 240 nominal species distributed in 18 genera (García et al. 2016), and 11 species distributed in five genera have been recorded from Japan (Tanaka 2016). Some species of the family are regarded as potentially or actually important plant pests (Danzig et al. 2008; Hambleton 1976; Kawai 2003; Williams 1996), and they are investigated from biological in addition to agricultural and/or economic perspectives.

Some genera and species placed in Rhizoecidae, especially those classified in the tribe Xenococcini, show very close trophobiotic relationships with ants that belong to the genus *Acropyga* (Hymenoptera: Formicidae: Formicinae) (Kishimoto-Yamada et al. 2005; LaPolla et al. 2002; LaPolla et al. 2008). Trophobiosis is commonly observed in interactions among many ants and honeydew-producing insects, including some scale insects, but the relationship between *Acropyga* ants and their trophobiont Rhizoecidae species is known as one of the most peculiar examples. Virgin queens of *Acropyga* ants grasping and carrying a root mealybug by their mandibles emerge from their nests for their mating flight and subsequent colony founding (LaPolla et al. 2002; LaPolla et al. 2008), the mealybugs are maintained in the ant’s nest (Flanders 1957; Kishimoto-Yamada et al. 2005; LaPolla et al. 2002), and the ants feed on the honeydew excreted by the mealybugs, which in turn feed on root-sap (Johnson et al. 2001; Smith et al. 2007). This obligatory relationship has greatly interested many researchers, and as a result mealybugs and other scale insects associated with *Acropyga* ants have been relatively well studied systematically and/or taxonomically (LaPolla et al. 2008; Maruyama et al. 2013; Schneider and LaPolla 2011; Williams 1998; Williams 2004; Williams and LaPolla 2004; Williams and Terayama 2000). Nevertheless, a mutualistic partner or associate of a rare Japanese ant species, *Acropyga yaeyamensis*, have not been hitherto detected nor described.

Recently, the author had an opportunity to examine several interesting mealybug specimens that were collected from the nest of *Acropyga yaeyamensis* which showed a close association with the ant (Figure 2), being a potential mutualistic trophobiont. The species is quite peculiar and distinctive among known Rhizoecidae species, although it is somewhat similar to *Capitisetella migrans* and *Pseudorhizoecus proximus*.

This paper describes and illustrates this mealybug as a new genus and species on the basis of adult female morphology. A key to Japanese species of Rhizoecidae is also given.

## Materials and methods

Specimens of the root mealybug species used in this study were originally collected by a collaborator of this study, Mr. Taku Shimada, on 16 February 2015, from a nest of *Acropyga yaeyamensis* Terayama & Hashimoto, 1996, in Mt. Omoto, Ishigaki Is., Japan. The mounting method used for these specimens followed that described by Tanaka (2014). Morphology of the slide-mounted specimens was examined under a phase-contrast light microscope (Olympus BH2-PH). The terminology used to describe the adult females followed that of Kozár and Konczné Benedicty (2007). All examined type materials in this study are deposited in the National Museum of Nature and Science, Tsukuba, Japan (NSMT).

## Taxonomy

### Tribe Rhizoecini

Taxon classificationAnimaliaHemipteraRhizoecidae

Williams, 1969

#### Type genus.

*Rhizoecus* Künckel d’Herculais, 1878

#### Diagnosis.

Same as for the family, except the characters related to Xenococcini (adopted and modified from Kozár and Konczné Benedicty 2007).

### Subtribe Ripersiellina

Taxon classificationAnimaliaHemipteraRhizoecidae

Kozár, 2007

#### Type genus.

*Ripersiella* Tinsley, 1899 in Cockerell 1899

#### Diagnosis.

Same as the family, except the characters related to Xenococcini, Geococcina and Rhizoecina. Most of the species have bitubular pores, and 5–6 segmented antennae. In some monotypic genera, included tentatively, the anal ring and/or the antennae are reduced (adopted and modified from Kozár and Konczné Benedicty 2007).

### Ishigakicoccus gen. n.

Taxon classificationAnimaliaHemipteraRhizoecidae

http://zoobank.org/0F3B70B0-9BF3-4C46-B9D8-0DC17AC12C5F

Figures 1 , 2 , 3

#### Type species.

*Ishigakicoccus shimadai* sp. n., here designated.

#### Diagnosis.

Body elongate oval, slightly pyriform. Eye spot absent. Antennae 5 segmented. Legs well-developed. Two types of body setae present on both dorsal and ventral body surface. Small sized multilocular pores present, each with 5–6 loculi. Tritubular and bitubular pores absent. Oral collar tubular ducts absent. Ostioles absent. Anal ring irregular oval; anal pores and anal ring setae absent. Large 3–5 locular pore without central hub, present on medial area of ventral abdomen. A circulus present.

#### Etymology.

Named after the island (*Ishigaki*) where the type species was collected first. The suffix *coccus* is commonly used in naming Coccomorpha genera. Gender: masculine.

**Figure 1.**
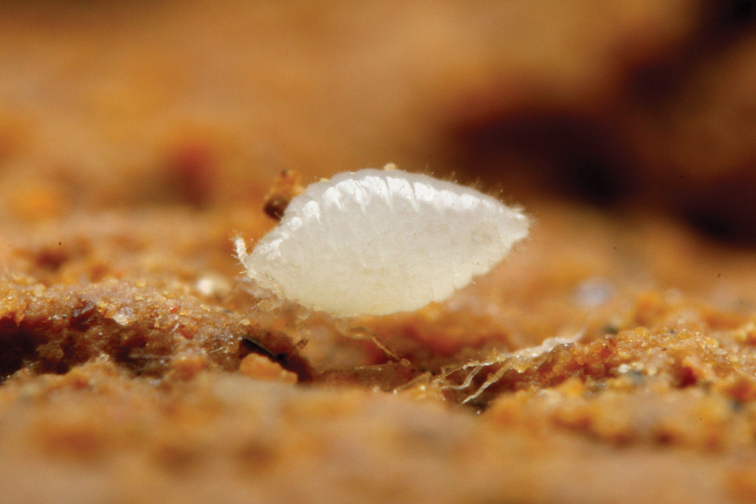
A mature adult female of *Ishigakicoccus shimadai* sp. n.

**Figure 2.**
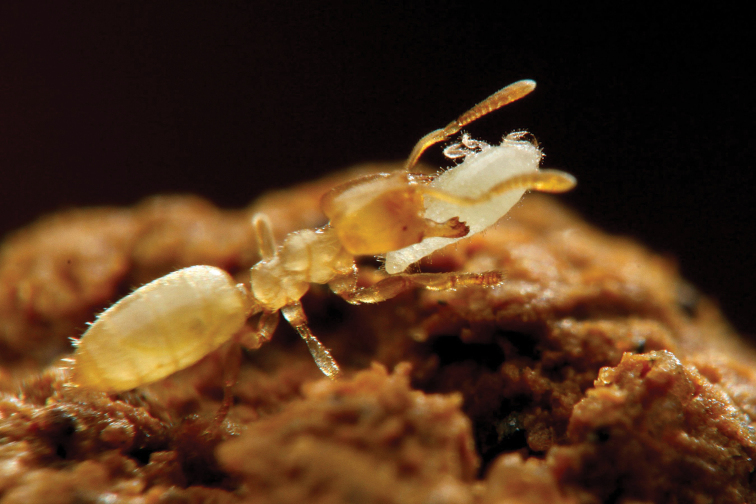
An adult female of *Ishigakicoccus shimadai* sp. n. being carried by *Acropyga yaeyamensis*.

**Figure 3.**
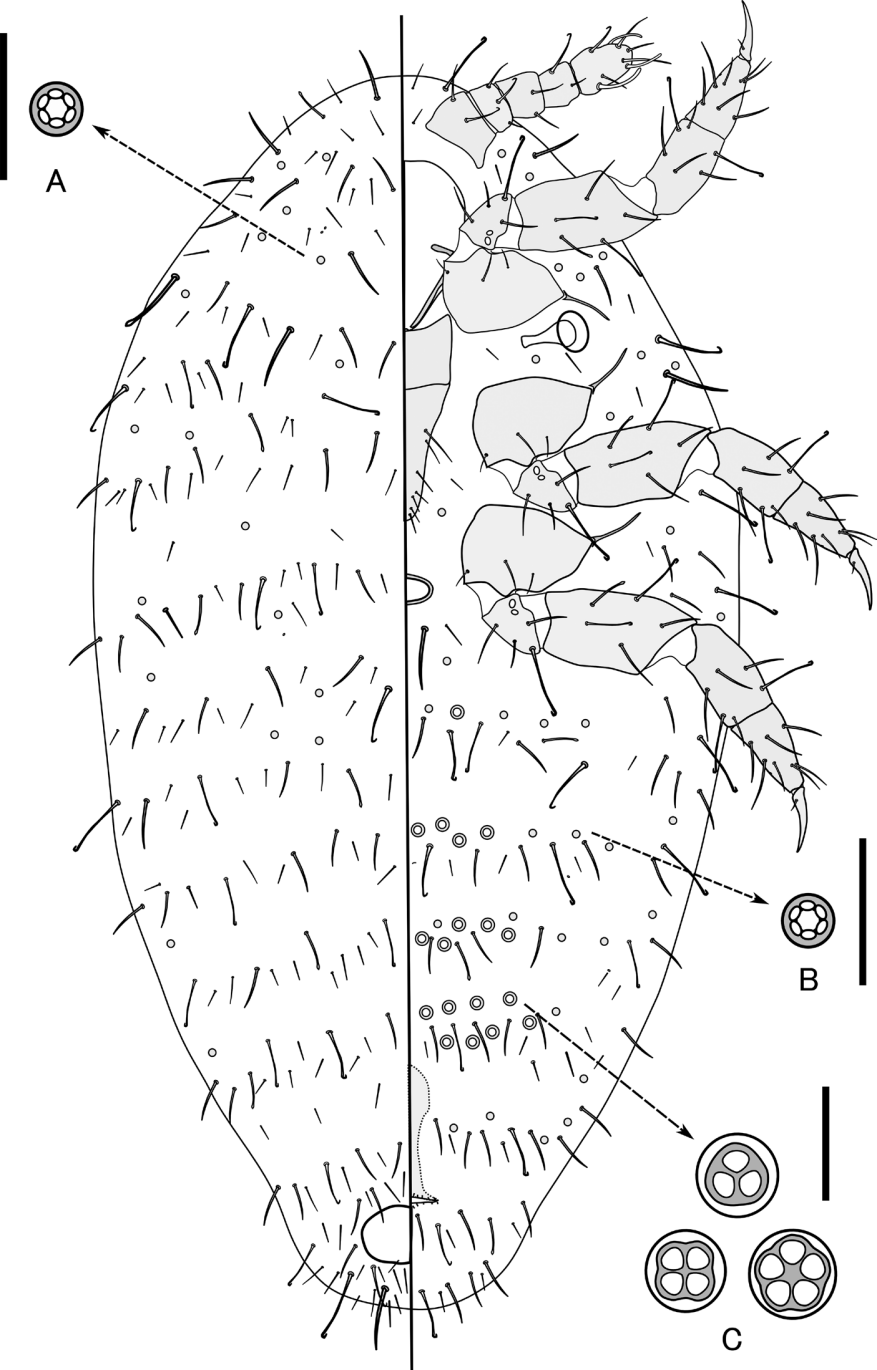
*Ishigakicoccus shimadai* Tanaka, sp. n. adult female. **A** Small multilocular pore on dorsal surface **B** Small multilocular pore on ventral surface **C** 3–5 locular pores on medial area of venter. Scale bars: 10 µm.

### Ishigakicoccus shimadai sp. n.

Taxon classificationAnimaliaHemipteraRhizoecidae

http://zoobank.org/A2DC112B-D63E-4D50-A0BB-080822CDF0CB

Figures 1 , 2 , 3

#### Type series.

Holotype, adult female. Japan, Okinawa-pref., Ishigaki Is., Mt. Omoto, 16.II.2015. coll. T. Shimada 1♀ (NSMT-I-Ho 00073). Paratypes, same data as Holotype, 3♀ (NSMT-I-Ho 00074–76).

#### Slide-mounted specimens.

Body elongate oval, slightly pyriform, 721–895 µm long, 379–632 µm wide. Eye spot absent. Antennae 5 segmented, 1^st^ : 18.4–29.5 µm long, 2^nd^ : 13.7–18.4 µm long, 3^rd^ : 15.8–26.3 µm long, 4^th^ : 17.1–22.1 µm long and 5^th^ : 36.8–41.3 µm long. The fifth segment with three fleshy setae. Anterior spiracles each 20.8–28.7 µm wide across atrium; posterior spiracles each 16.8–29.7 µm wide across atrium. Legs well developed, length of posterior pair: coxa 45.8–55.2 µm long, trochanter + femur 123–133 µm long, tibia + tarsus 112–122 µm long, claw 33.4–39.4 µm long. Ratio of lengths of tibia + tarsus to trochanter + femur 1.05–1.11. Claw digitules setose, each 3.2–6.1 µm long.

*Dorsum*: Two types of body setae present. Type I: relatively well developed setae, 19.7–58.9 µm long, some setae with a bent or rarely with a knobbed apex. Type II: relatively slender setose setae, 11.0–32.8 µm long, each with a pointed apex. Both types of setae distributed in transverse segmental rows. Multilocular pores each about 2.9–3.7 µm in diameter, each with 5–6 loculi, present on head and thorax, but rarely present on abdomen (Figure 3). Tritubular and bitubular pores absent. Oral collar tubular ducts absent. Ostioles absent. Anal opening 38.9–52.6 µm long and 55.2–61.8 µm wide. Anal ring irregular oval. Anal pores, and anal ring setae absent, but type I and II setae densely present around the opening.

*Venter*: Labium appearing 2 segmented, 100–107 µm long, 48.4–62.0 µm wide. Two types of body setae present: type I, relatively well developed setae, 18.4–65.3 µm long, some setae with a bent and a rarely knobbed apex.; type II, relatively slender setose setae, 10.5–37.4 µm long, each with a pointed apex. Both setae distributed in transverse segmental bands. Multilocular pores each about 2.9–3.7 µm in diameter, each with 5–6 loculi, frequently present and evenly distributed on all areas of venter. 3–5 locular pores, each 5.8–7.9 µm in diameter, without central hub, present on medial area of abdominal segments III–VI in transverse rows. A circulus, 10.5–18.4 µm long and 21.0–33.2µm wide present on medial anterior border of abdominal segment II. Tritubular and bitubular pores absent. Oral collar tubular ducts absent.

#### Etymology.

Named after the collector of the type specimens.

#### Host plant.

Unknown.

### Identification key to Japanese Rhizoecidae species (adopted and modified from Kozár and Konczné Benedicty 2007, Schneider and LaPolla 2011)

**Table:** 

1	Ostioles present	**2**
–	Ostioles absent	**9**
2	Anal lobes well-developed, with stout spine-like setae	**3 (Genus *Geococcus*)**
–	Anal lobes not well-developed, without stout spine-like setae	**4**
3	Stout, spine-like setae on dorsum present. Multilocular pores with 7–10 loculi, present on ventral surface (around vulva)	***Geococcus oryzae* (Kuwana, 1907)**
–	Stout, spine-like setae on dorsum absent. Multilocur pores with 6 loculi present on all segments of venter and most segments of dorsum	***Geococcus citrinus* Kuwana, 1923**
4	Tritubular pores present. Bitubular pores absent	**5 (Genus *Rhizoecus*)**
–	Tritubular duct absent. Bitubular pores present	**6 (Genus *Ripersiella*)**
5	Multilocular pores absent on venter	***Rhizoecus cacticans* (Hambleton, 1946)**
–	Multilocular pore present on ventral surface of posterior abdominal segments	***Rhizoecus advenoides* Takagi & Kawai, 1971**
6	Multilocular pores absent on venter	***Ripersiella sasae* (Takagi & Kawai, 1971)**
–	Multilocular pores present at least on ventral surface of posterior abdominal segments	**7**
7	Multilocular pores present on venter and dorsum (at least on medial part of two segments of dorsum)	***Ripersiella hibisci* (Kawai & Takagi, 1971)**
–	Multilocular pores pores only on venter (often some on dorsum, but only on medial part of segment VII)	**8**
8	With two circuli	***Ripersiella kondonis* (Kuwana, 1923)**
–	With one circulus	***Ripersiella theae* (Kawai & Takagi, 1971)**
9	Disc pores present on venter and dorsum. Antennae 5 segmented	***Ishigakicoccus* gen. n. *shimadai* sp. n**.
–	Disc pores absent. Antennae with 2 to 4 segments	**10**
10	Antennae 4 segmented	***Xenococcus kinomurai* (Williams & Terayama, 2000)**
–	Antennae 2 segmented	**11 (Genus *Eumyrmococcus*)**
11	Circulus absent. Body with flagellate setae only, sensory setae absent on body surface	***Eumyrmococcus smithii* Silvestri, 1926**
–	A small circulus present on abdominal segment III. Sensory setae present on body surface	***Eumyrmococcus nipponensis* Terayama, 1986**

## Discussion

The genus and species described in this paper slightly resembles two other species of the monotypic genera of Rhizoecidae, *Capitisetella migrans* (Green, 1933) and *Pseudorhizoecus proximus* Green, 1933, in lacking ostioles and tritubular and/or bitubular pores, and having a pyriform to round body shape. However, the former is clearly distinguishable from those two species by having small multilocular pores on both surfaces of the body, 3–5 large locular pores on the medial area of the ventral abdomen, and two different-sized body setae. These morphological differences are here considered as good for the establishment of a new genus. Based on some morphological similarity, the genus and species is tentatively placed in the subtribe Ripersiellina Kozár 2007 of the tribe Rhizoecini Williams, 1969, in which *Capitisetella migrans* and *Pseudorhizoecus proximus* are also placed (Kozár and Konczné Benedicty 2007).

In east and southeast Asia, many species in tribe Xenococcini have been frequently found in *Acropyga* ants’ nests (Williams 1998), and the xenococcine species appear to be the main mutualistic partners of *Acropyga* ants. However, the new species described in this paper is clearly different from all of them in having small multilocular pores, 3–5 locular pores, and 5 segmented antennae. Although the exact phylogenetic position of this new species is still unclear, but morphological features of the specimens indicate that this species apparently does not belong to tribe Xenococcini.

The collector of the species, Mr. Taku Shimada, observed some *Acropyga yaeyamensis* workers grabbing and carrying the root mealybug individuals by their mandibles when he collected the species (Figure 2). This behavioral observation, the collecting site of the species, i.e. inside a nest of *Acropyga yaeyamensis* and the morphological features of the species, i.e. the lack of wax on the body surface (Figure 1 and Figure 2), which is usually observed in most myrmecophilous scale insects (LaPolla et al. 2008) indicates that the species is a potential trophobiont of *Acropyga yaeyamensis*, even though it does not appear to be a xenoccocine species. Similar associations between *Acropyga* ants and scale insects that are not members of the tribe Xenococcini have been sometimes, but not commonly, observed in the neotropics, the United States, and Australia (LaPolla et al. 2002; LaPolla et al. 2008; Jonson et al. 2001; Williams 1998; Williams and LaPolla 2004). One of the most extraordinary cases was observed in Australia; an ortheziid scale insect, *Acropygorthezia williamsi* LaPolla & Miller, 2008, which belongs to the family Ortheziidae, not Rhizoecidae, showed a close trophobiotic association with *Acropyga myops* (LaPolla et al. 2008). Intensive molecular phylogenetic analysis of scale insects having trophobiotic associations with *Acropyga* ants, including non-xenococcine trophobiont and/or potential trophobiont species of *Acropyga* ants, related co-evolution and co-speciation analyses and behavioral ecological studies may provide new insights into the evolution and maintenance of this unique obligate mutualism.

## Supplementary Material

XML Treatment for Rhizoecini

XML Treatment for Ripersiellina

XML Treatment for Ishigakicoccus

XML Treatment for Ishigakicoccus shimadai
